# 2-Amino-4-(nitroalkyl)-4H-chromene-3-carbonitriles as New Cytotoxic Agents 

**Published:** 2013

**Authors:** Afsaneh Zonouzi, Roghieh Mirzazadeh, Maliheh Safavi, Sussan Kabudanian Ardestani, Saeed Emami, Alireza Foroumadi

**Affiliations:** a *School of Chemistry, University College of Science, University of Tehran, P.O. Box 14155- 6455, Tehran, Iran.*; b *Faculty of Pharmacy and Pharmaceutical Sciences Research Center, Tehran University of Medical Sciences, Tehran, Iran. *; c *Institute of Biochemistry and Biophysics, Department of Biochemistry, University of Tehran, Tehran, Iran. *; d *Department of Medicinal Chemistry and Pharmaceutical Sciences Research Center, Faculty of Pharmacy, Mazandaran University of Medical Sciences, Sari, Iran****. ***; e *Drug Design and Development Research Center, Tehran University of Medical Sciences, Tehran, Iran. *

**Keywords:** 4*H*-chromenes, Benzopyran, DBU, One-pot synthesis, Cytotoxic activity

## Abstract

A series of 2-amino-4-(nitroalkyl)-4*H*-chromene-3-carbonitriles were synthesized by an efficient multicomponent reaction in aqueous media using DBU as a catalyst at room temperature. Mild condition, environment friendly procedure and excellent yields are the main advantages of this procedure. The cytotoxic activity of target compounds were evaluated against three cancer cell lines MDA-MB-231, MCF-7 and T47D in comparison with etoposide as reference drug. Generally, all compounds showed good cell growth inhibitory activity with IC^50^ values less than 30 μg/mL. Their activities were comparable or more potent than standard drug etoposide. The 6-bromo- derivatives 7e and 7f showed promising cytotoxic activity with IC50 values in the range of 3.46–18.76 μg/mL, being more potent than etoposide against all tested cell lines.

## Introduction

**Figure 1:**
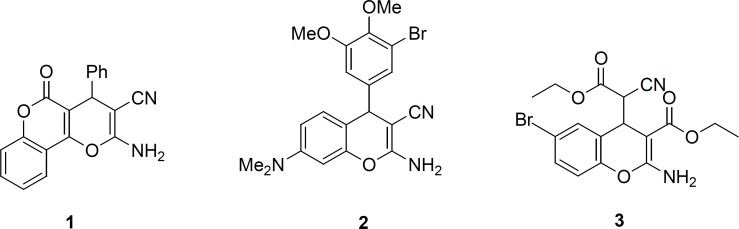
Structures of some 2-amino-4*H*-chromenes with diverse biological activities

4*H*-Chromene derivatives are important scaffold in organic and medicinal chemistry. They belong to a class of naturally occurring benzopyran derivatives with a wide range of biological applications, such as antiallergic (1), anti-proliferative (2), anticancer (3, 4), antibacterial (5, 6), antiviral (7) and potent apoptosis inducers (8). Such diverse biological activities have made chromene derivatives important for further development in medicinal and organic synthesis studies (9, 10). In particular, 2-amino-4*H*-chromenes are of recent interest for their cytotoxic activities (11, 12); other biological activities have been observed, for instance, pyranopyranone 1 that served as precursor for the blood anticoagulant warfarin (13), benzopyrane 2 has been known for anticancer therapeutic (14) and(4*H*-chromen-4- yl) cyanoacetate 3 as inhibitor of Bcl-2 protein and apoptosis inducer (Figure 1) (15). 

Multicomponent reactions have been successfully employed to generate highly diverse combinatorial libraries for high-throughput screening of biological and pharmacological activities (16, 17). This type of reaction becomes increasingly important in organic and medicinal chemistry because it allows obtaining highly sophisticated polyfunctional molecules through simple one-pot procedures (18, 19). Multicomponent reaction protocol with environmentally benign solvents and catalytic systems is one of the most suitable strategies, which meets the requirements of green aspects of chemistry for developing libraries of medicinal scaffolds. Developing organic reactions in water has become highly popular in recent years due to its specific properties in mediating organic reactions and its friendliness to the environment (20-22).

Several procedures for the multicomponent preparation of 2-amino-4*H*-chromenes have been described (23-25). Earlier Elinson et al. have reported synthesis of 2-amino-4*H*-chromenes in the presence of NaOAc or KF as base (26). This method has been directed to offer corresponding 4*H*-chromenes by using malononitrile or cyanoacetate as one of C-H acids and exchange of substitution on 4-position. 

Thus, considering the fact that the discovery of a novel anticancer drug is a urgent need (27, 28), in continuation of our research program to find a novel anticancer agent (29, 30), we developed a general rapid, easy and environmentally benign synthetic protocol for the synthesis of functionalized chromenes bearing 4-nitroalkyl moiety instead of the 4-aryl ring of cytotoxic agents 2-amino-4-aryl-4*H*-chromene-3-carbonitriles.

## Experimental

*Chemistry*

Chemicals and solvents were obtained from Merck (Germany) and Fluka (Switzerland) and were used without further purification. Column chromatography was performed on silica gel (0.015-0.04 mm, mesh-size) and TLC on precoated plastic sheets (25 DCUV-254). Melting points were measured on a Barnstead Electrothermal melting point apparatus and are not corrected. Elemental analyses for C, H and N were performed using a Thermo Finnigan Flash EA1112 instrument. IR spectra were recorded on a Shimadzu FT-IR-4300 spectrophotometer as KBr discs. 1H NMR spectra were determined in CDCl_3_ on a Brucker 500 spectrophotometer and chemical shifts are expressed in ppm downfield from tetramethylsilane. Mass spectra were recorded on a Finnigan-MAT 8430 spectrometer at an ionization potential of 70 ev.

*General procedure for the synthesis of 2-amino-4-(nitroalkyl)-4H-chromene-3-carbonitrile derivatives (7a–f)*

To a magnetically stirred mixture of salicylaldehyde 4 (1 mmol), malononitrile 5 (1 mmol) and nitroalkane 6 (2 mmol) in water (5 mL), 1,8-diazabicyclo[5.4.0]undec-7-ene (DBU, 30 mol%) was added. The reaction mixture was stirred for 6 h at room temperature. The mixture was extracted with EtOAc (6 mL) and the organic phase was dried over Na2SO4, concentrated and purified on a silica gel column using EtOAc/hexane (4:1) as eluent to afford the product 7.

*2-Amino-4-(nitromethyl)-4H-chromene-3-carbonitrile (7a)*

White powder; yield 85%; m.p. 138- 139 °C (Lit. 139-140 °C) (25); 1H NMR (500 MHz, CDCl_3_): 4.29 (dd, *J *= 6.7 and 4.7 Hz, 1H), 4.50 (dd, *J *= 12.5 and 6.8 Hz, 1H), 4.60 (dd, *J *= 12.5 and 4.7 Hz, 1H), 5.57 (br s, 2H), 6.9 (d, *J *= 8.7 Hz, 1H), 7.29-7.30 (m, 2H), 7.40 (dd, *J *= 8.7 and 2.3 Hz, 1H). 

*2-Amino-4-(1-nitroethyl)-4H-chromene-3-carbonitrile (7b)*

White powder; yield 80%; diastereomeric ratio 1.2:1; m.p. 165-166 ºC; major diastereomer: ^1^H NMR (500 MHz, CDCl_3_): 1.60 (d, *J *= 6.5 Hz, 3H), 4.2 (d, *J *= 6.5 Hz, 1H), 4.54-4.56 (m, ^1^H), 4.92 (br s, 2H), 6.98-7.35 (m, 4H); minor diastereomer: ^l^H NMR (500 MHz, CDCl_3_): 1.37 (d, *J *= 6.5 Hz, 3H), 4.4 (d, *J *= 3.5 Hz, 1H), 4.71-4.73 (m, ^1^H), 4.88 (br s, 2H), 6.98-7.35 (m, 4H); IR (KBr): 3429, 3324 (NH_2_), 3027, 2993 (CH), 2201 (CN), 1604, 1571 (C=C), 1558 and 1381 (NO_2_); MS (m/z): 245 (M^+^), 218 (M^+^-HCN), 215 (M^+^-NO), 199 (M^+^-NO_2_), 172 (M+-NO_2_, HCN), 144 (M^+^-HCN, CH_3_CHNO_2_), 114 (C_9_H^6+^), 77, 57. Anal. Calcd (%) for C_13_H_13_N_3_O_3_: C, 58.77; H, 4.52; N, 17.13. Found: C, 58.72; H, 4.45; N, 17.17. *2-Amino-6-chloro-4-(nitromethyl)-4H-chromene-3-carbonitrile (7c) *

Yellow powder; yield 83%; m.p. 181-182 ºC; 1H NMR (500 MHz, CDCl_3_,): 4.33 (t, 1H), 4.55 (dd, *J *= 12.5 and 6.5 Hz, 1H), 4.63 (dd, *J *= 12.8 and 4.0 Hz, 1H), 4.84 (br s, 2H), 7.0 (d, *J *= 8.5 Hz, 1H), 7.18 (d, *J *= 2.5 Hz, 1H), 7.28-7.30 (m, 2H), 7.40 (dd, *J *= 8.7 and 2.3 Hz, 1H). 

*2-Amino-6-chloro-4-(1-nitroethyl)-4H-chromene-3-carbonitrile (7d)*

Yellow powder; yield 75%; diastereomeric ratio 1:1; m.p. 166–167 ºC; 1H NMR (500 MHz, CDCl_3_): 1.42 (d, *J *= 7.0 Hz, 3H), 1.60 (d, *J *= 6.5 Hz, 1H), 4.2 (d, *J *= 6.5 Hz, 1H), 4.36 (d, *J *= 3.5 Hz, ^1^H), 4.54-4.56 (m, ^1^H), 4.70-4.72 (m, 1H), 4.88 (br s, 2H), 6.99-7.31 (m, 4H); MS (m/z): 279, 281 (M^+^, M^++2^), 233, 235 (M^+^, M^++2^-NO_2_), 205, 207 (M^+^, M^++2^-CH_3_-CH-NO_2_), 171, 179 (M^+^-HCN, CH_3_CHNO_2_), 114 (C_9_H^6+^), 77, 57. Anal. Calcd (%) for C_12_H10ClN3O3: C, 51.52; H, 3.60; N, 15.02. Found: C, 51.49; H, 3.64; N, 15.07. 

*2-Amino-6-bromo-4-(nitromethyl)-4H-chromene-3-carbonitrile (7e)*

White powder; yield 85%; m.p. 192-193 ºC; 1H NMR (500 MHz, CDCl3): 4.39 (t, 1H), 4.51 (dd, *J *= 13.75 and 7.0 Hz, 1H), 4.63 (dd, *J *= 12.5 and 4.5 Hz, 1H), 4.85 (br s, 2H), 7.0 (d, *J *= 8 Hz, 1H), 7.17-7.20 (m, 2H), 7.33 (t, 1H); IR (KBr): 3440, 3326 (NH2), 3030, 2998 (CH), 2204 (CN), 1608, 1574 (C=C), 1572, 1377 (NO_2_), 812. 

*2-Amino-6-bromo-4-(1-nitroethyl)-4H-chromene-3-carbonitrile (7f)*

White powder; yield 75%; diastereomeric ratio 1.6:1; m.p. 178-179 ºC; major diastereomer: ^1^H NMR (500 MHz, CDCl3): 1.60 (d, *J *= 7.0 Hz, 3H), 4.18 (d, *J *= 6.5 Hz, 1H), 4.54-4.56 (m, ^1^H), 4.89 (br s, 2H), 6.93-7.45 (m, 4H); minor diastereomer: lH NMR (500 MHz, CDCl_3_): 1.43 (d, *J *= 6.5 Hz, 3H), 4.35 (d, *J *= 3.5 Hz, 1H), 4.70-4.72 (m, 1H), 4.86 (br s, 2H), 6.93-7.45 (m, 4H); IR (KBr): 3432, 3325 (NH_2_), 3029, 2996 (CH), 2194 (CN), 1600 (C=C), 1542, 1386 (NO_2_), 820; MS (m/z): 322, 324 (M+, M^++2^), 263, 265 (M^+^, M^++2^-NO, HCN), 249, 251 (M^+^-NO_2_, HCN), 221, 223 (M^+^, M^++2^-HCN, CH_3_CHNO_2_), 170 (M^+^-HBr, CH_3_CHNO_2_), 143(M^+^-HCN, HBr, CH_3_CHNO_2_), 114 (C9H^6+^), 77, 57. Anal. Calcd (%) for C_12_H_10_BrN_3_O_3_: C, 44.47; H, 3.11; N, 12.96. Found: C, 44.51; H, 3.08; N, 12.99.

*Biological activity*

*Cell lines and cell culture*

The cell lines were purchased from the National Cell Bank of Iran (NCBI). The cells were grown in RPMI-1640 medium (GibcoeBRL, UK) supplemented with 10% heat-inactivated fetal calf serum (GibcoeBRL, UK) and 100 mg/mL streptomycin and 100U/mL penicillin at 37 ºC in a humidified atmosphere with 5% CO_2_ in air.

*In-vitro cytotoxicity assay*

The in vitro cytotoxic activity of each synthesized compounds 7a-f were assessed in comparison with etoposide using MTT colorimetric assay, first described by Mosmann (31) with modifications. Briefly, cultures in the exponential growth phase were trypsinized and diluted in complete growth medium to give a total cell count of 5×10^4^ cells/mL. 195 μl of suspension was added to wells of sterile 96-well plates (NUNC, Denmark) and allowed to attach overnight. The stock solutions of compounds were prepared in DMSO and then serially diluted with growth medium. After plating, 5 μL of a serial dilution of every compound was added. Each compound dilution was assessed in triplicate. The maximum amount of DMSO in the cell culture was 1%. Etoposide was used as positive control for cytotoxicity while three wells containing tumor cells cultured in 200 μL of complete medium were used as controls for cell viability. The plates were then incubated for 48 h. After incubation, 200 μL of RPMI-1640 without phenol red containing a final concentration of 0.5 mg/mL MTT (Sigma-Aldrich, Steinheim, Germany) was added to each well and the plate was incubated for another 4 h. After incubation, the culture medium was replaced with 100 μL of DMSO. Then the absorbance of each well was measured by using a microplate reader at 492 nm wavelengths. For each compound, the concentration causing 50% cell growth inhibition (IC_50_) compared with the control was calculated from concentration response curves by regression analysis. 

## Results and Discussion

*Chemistry*

We report in this paper, highly efficient one-pot synthesis of 2-amino-4-(nitroalkyl)- 4*H*-chromene derivatives 7a-f by using 1,8-diazabicyclo[5.4.0]undec-7-ene (DBU) as a catalyst in water at room temperature. A variety of bases with different pKa have been used for some multicomponent reactions. DBU base (pK_a_ = 12) is a neutral organic base with high basicity and has been used in many organic transformation in recent years (32). It is a sterically hindered amidine base and especially useful where side reactions due to the inherent nucleophilicity of basic nitrogen are a problem (33, 34). 

**Figure 2:**
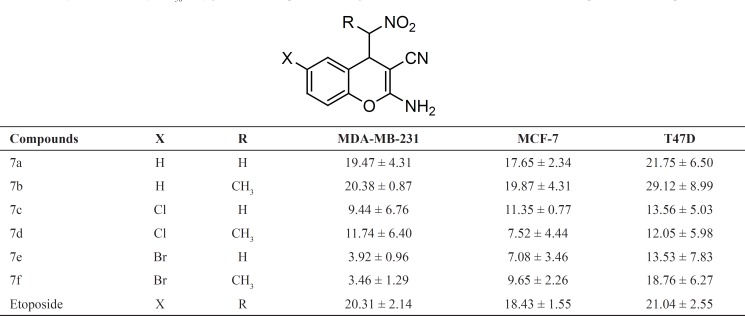
DBU-catalyzed one-pot, three-component synthesis of 2-amino-4-(nitroalkyl)-4*H*-chromene-3-carbonitriles

In our attempts to develop an efficient protocol, we focused on the efficient condensation of salicylaldehyde 4a, malononitrile 5 and nitromethane 6a in water at room temperature by several amounts of DBU as base (Figure 2). 

The best 85% yield of 2-amino-4-(nitromethyl)-4*H*-chromene-3-carbonitrile 7a was obtained when the reaction was performed using 30 mol% DBU at room temperature for 6 h. Under this optimal condition, salicylaldehydes 4, malononitrile 5 and nitroalkane 6 were condensed into corresponding substituted 2-amino-4- (nitroalkyl)-4*H*-chromenes 7a–f in 75-85% yields. The attempts with 3-, 4- or 5-methoxy derivatives of salicylaldehydes indicated that these aldehydes are not good candidates for this reaction. The 4-(nitroethyl)-products 7b, 7d and 7f were eluted from the column chromatography as mixtures of two diastereomers. The ratio of diastereomers was determined by 1 H NMR spectroscopy data. The IR spectrum of 7a exhibited bands at 3442, 3327, 2204, 1580 and 1420 cm^-1^ indicating the presence of NH_2_, CN and NO_2_ functionalities, respectively. The 1H NMR spectrum evidenced a characteristic of doublet of doublet at 4.29 ppm due to H-C_4_. 

**Figure 3:**
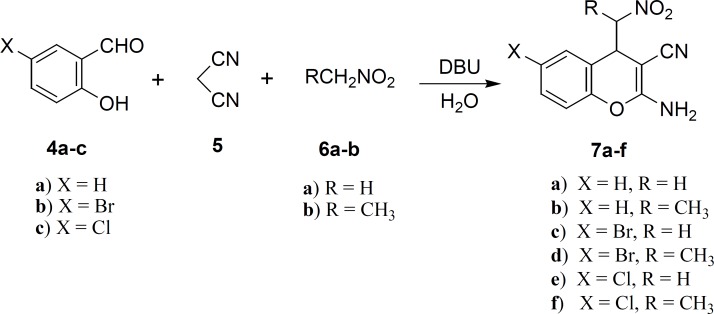
The proposed mechanism of DBU-catalyzed synthesis of 2-amino-4-(nitroalkyl)-4*H*-chromene-3-carbonitriles

According to the proposed mechanism is shown in Figure 3, the first step of this reaction is the formation of Knoevenagel product 8 by the condensation of salicylaldehyde with malononitrile (5) acting DBU as base. Two subsequent reaction pathways are possible for Knoevenagel product 8. One pathway (A) includes the Michael addition of nitroalkane 6 to the Knoevenagel adduct 8 followed by intramolecular cyclization leads to corresponding 2-amino-4-(nitroalkyl)-4*H*-chromenes 7 and another pathway (B) involves DBU accelerated intramolecular cyclization of Knoevenagel adduct 8 into reactive 2-imino-2*H*-chromene 9 with a subsequent DBU promoted Michael addition of nitroalkane 6. 

*Cytotoxic activity*

The cytotoxic activity of synthesized compounds 7a-f was tested in vitro against cancer human cell lines including MDA-MB-231, MCF-7 and T47D. The assays were performed in 96-well plates using MTT colorimetric method in comparison with etoposide (31). For each compound, the concentration causing 50% cell growth inhibition (IC_50_) compared with the control was calculated from concentration response curves by regression analysis. The results are summarized in Table 1.

**Table 1:** Cytotoxic activity (IC_50_, in μg/mL)a of compounds7a-f against three cancer human cell lines in comparison with etoposide

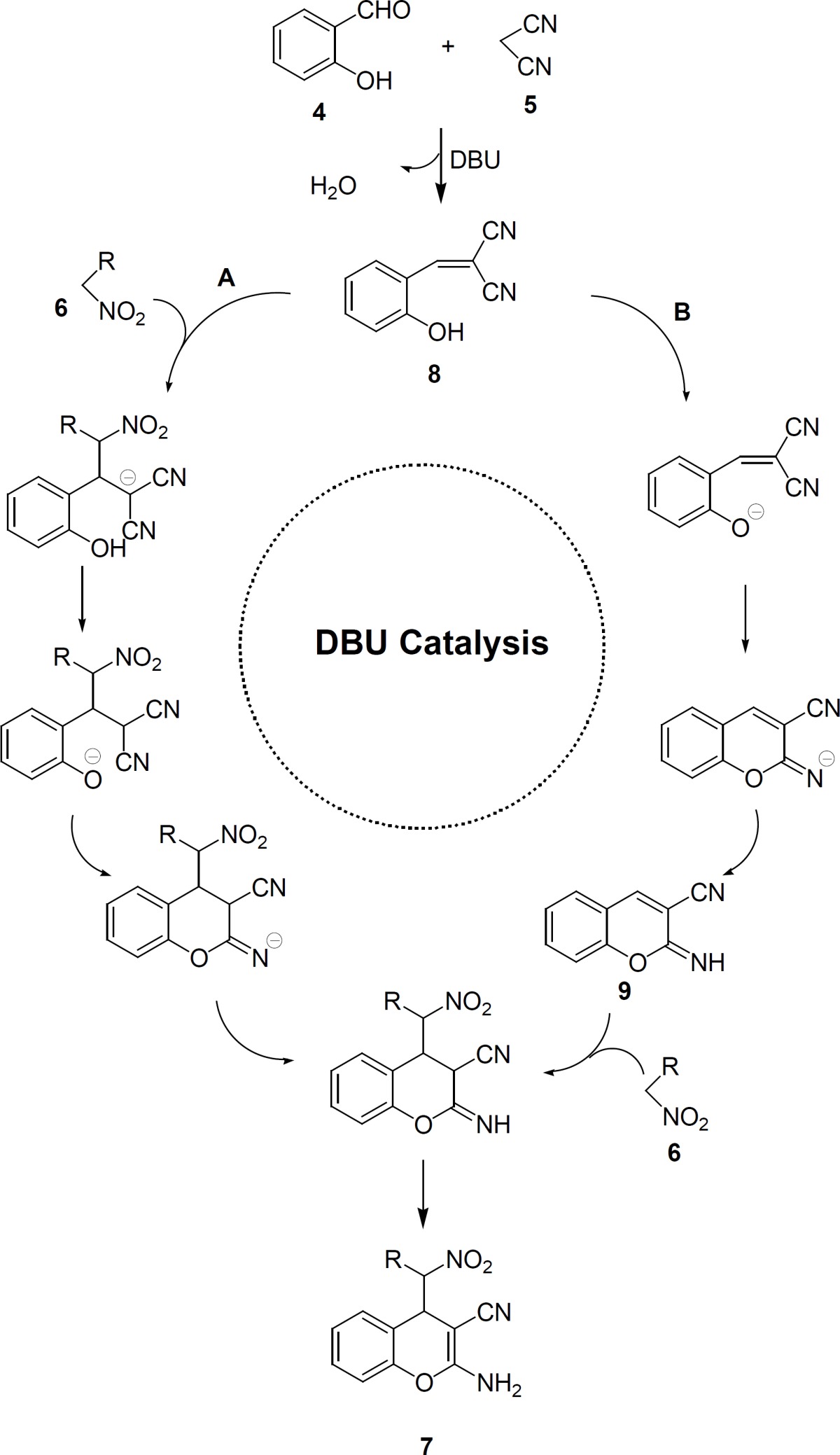

^a ^The IC_50_ values represent an average of three independent experiments (mean ± SD)

Generally, all compounds showed good cell growth inhibitory activity with IC_50_ values less than 30 μg/mL. Moreover, the susceptibilities of MDA-MB-231 and MCF-7 cells to target compounds 7a-f was more than T47D cell line. Among the test compounds, 6-Br derivatives 7e and 7f showed more potent activity against MDA-MB-231 cell line (IC_50_< 4 μg/mL). In this case, their activity was 4-times more than standard drug etoposide. In addition, compounds 7c and 7d exhibited higher growth inhibitory activity against MDA-MB-231 cell line in comparison with etoposide. The IC_50 _values of test compounds against MCF-7 revealed that all compounds showed equipotent or more potent activity respect to the etoposide. Among them, the highest activity was observed with 7d and 7e, being 2-fold more potent than standard drug. By comparing the IC_50_ of compounds 7a-f with that of etoposide against T47D, in could be concluded that 6-substituted chromene derivatives 7c-f exhibit better activity. The activity of compound 7a against T47D cells was comparable to etoposide. The biological evaluation of this limited series of 4-(nitromethyl)-4*H*-chromenes demonstrated that the introduction of halogen at the 6-position of the chromene ring increases cytotoxic activity. Furthermore, the insertion of α-methyl respect to the nitro group, could not improve cytotoxic activity meanwhile introduced a new chiral centre and produced diastereomeric mixture.

## Conclusion

In conclusion, we have developed a facile, convenient and environmentally benign one-pot synthesis of multi-substituted chromenes using DBU as a catalyst in aqueous media at room temperature. In this instance, it is possible to apply the tenets of green chemistry to a medicinal setting in the development of new methodology to the biologically interesting molecules. Furthermore this strategy involves additional advantage over previous methods for proceeding under easy and mild conditions. All synthesised compounds showed good cytotoxic activity against MDA-MB-231, MCF-7 and T47D cell lines. Their activities were comparable or more potent than standard drug etoposide. These results indicate that 2-amino- 4*H*-chromene-3-carbonitriles containing 4-(nitroalkyl)- moiety could serve as a new basis for the development of novel group of anticancer agents. 
